# Recurrent Merkel Cell Carcinoma Following COVID-19 Treatment

**DOI:** 10.7759/cureus.64863

**Published:** 2024-07-18

**Authors:** Hirofumi Kawamoto, Natsuko Saito-Sasaki, Yu Sawada

**Affiliations:** 1 Dermatology, University of Occupational and Environmental Health, Kitakyushu, JPN

**Keywords:** histology, tumor, case report, covid-19, merkel cell carcinoma

## Abstract

Merkel cell carcinoma (MCC) is an aggressive neuroendocrine skin cancer influenced by the immune system. Recent studies suggest that viral infections, notably COVID-19, may exacerbate such malignancies. This case report explores potential mechanisms by which SARS-CoV-2, the virus responsible for COVID-19, could influence the behavior and proliferation of malignant tumor cells. Emerging evidence indicates that COVID-19 may disrupt immune surveillance and modulation, which are critical in controlling the spread and severity of cancers, including MCC. Additionally, the cytokine storm induced by COVID-19 is proposed to facilitate tumorigenic activity, potentially enhancing MCC aggressiveness. By integrating clinical findings with contemporary immunological and virological theories, this report aims to contribute to the understanding of COVID-19's impact on cancer progression, specifically MCC, emphasizing the need for comprehensive management strategies in cancer patients during the pandemic.

## Introduction

Merkel cell carcinoma (MCC) is an aggressive neuroendocrine skin cancer whose progression is influenced by the immune system's status [1]. It is known as the most aggressive cutaneous malignancy due to its high potential for invading the dermal-lymphatic system and spreading to lymph nodes and other parts of the body through the bloodstream. Contributing factors to its development include sunlight exposure and weakened immune function [2]. MCC shares several characteristics with small-cell lung cancer, including sensitivity to radiation and chemotherapy, as well as a strong tendency for aggressive metastasis. Optimal treatment outcomes are achieved through early diagnosis and a combination of surgery, radiation therapy, and chemotherapy. Recent observations suggest that viral infections, notably COVID-19, may play a pivotal role in the exacerbation of such malignancies [3]. This case report aims to examine the potential mechanisms by which SARS-CoV-2, the virus responsible for COVID-19, could influence the proliferation and behavior of malignant tumor cells. Emerging evidence suggests that COVID-19 may disrupt immune surveillance and modulation, which are crucial in controlling the spread and severity of cancers, including MCC. Additionally, the cytokine storm induced by COVID-19 has been proposed as a facilitator of tumorigenic activity, possibly enhancing the aggressiveness of MCC [4]. By integrating clinical findings with contemporary immunological and virological theories, this report endeavors to contribute to the understanding of how COVID-19 could impact cancer progression, with a specific focus on MCC, thus underscoring the imperative for comprehensive management strategies in cancer patients during the ongoing pandemic.

## Case presentation

Elderly individuals, particularly those with a history of previous malignancies, are at an increased risk of disease relapse of MCC [1]. Additionally, an immunosuppressed state can significantly influence the likelihood of recurrence. We introduce an 85-year-old male, with a medical history notable for prostate cancer, gallstones, ascending colon cancer, bladder cancer, and a chronic smoking habit of 20 cigarettes per day, who reported a new-onset tumorous skin rash on the inner side of his left upper arm. The patient, who had also received four doses of the COVID-19 vaccine (Pfizer COVID-19 vaccine BNT162b2), sought evaluation from a dermatologist. A skin biopsy was performed, revealing a proliferation of atypical lymphocytes within the dermis, raising suspicions of Merkel cell carcinoma (MCC).

**Figure 1:**
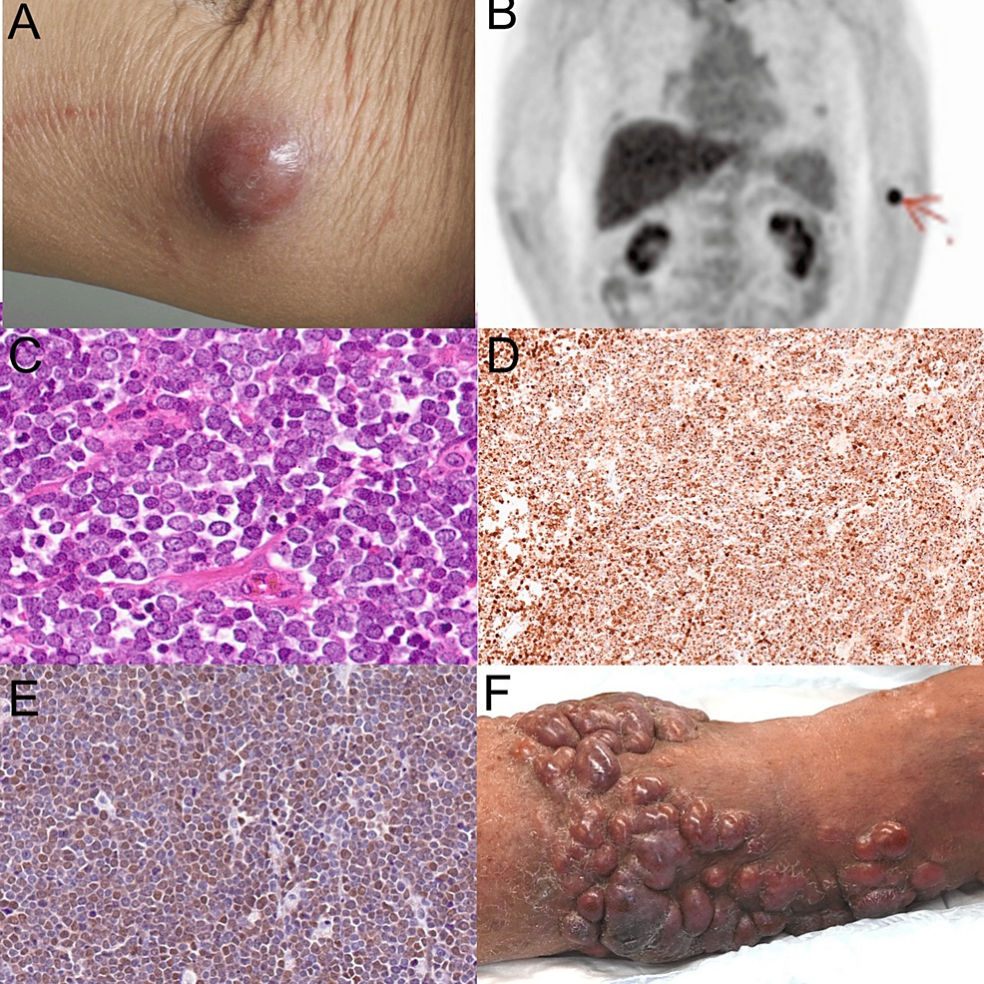
Clinical manifestation and histological examination (A) Clinical manifestation of the tumor at the first visit: The tumor appears as a 24×23 mm dome-shaped, firm, red lesion on the patient's left upper arm, demonstrating good mobility relative to the subcutaneous tissue. (B) PET-CT imaging: The scan shows increased metabolic activity in the left upper arm, indicating localized disease without evidence of distant metastasis. (C) H&E staining: Histopathological examination of the excised tumor reveals nodular growths in the subcutaneous tissue composed of cells with pale cytoplasms and inconspicuous nucleoli, characteristic of MCC. (D) Immunostaining for CK20: The staining shows a characteristic dot-like pattern, confirming the presence of MCC cells. (E) Immunostaining for Merkel Cell Polyomavirus. (F) A clinical manifestation of the recurrence of the tumors. PET-CT: positron emission tomography-computed tomography, H&E: hematoxylin and eosin, MCC: Merkel cell carcinoma, CK20: cytokeratin 20

During his initial examination, the dermatological assessment revealed a 24×23 mm dome-shaped, firm, red tumor with good mobility relative to the subcutaneous tissue (Figure 1A). Laboratory investigations showed mild renal impairment, an elevated HbA1c indicating poor glycemic control, and an increased level of neuron-specific enolase (NSE), a marker often elevated in neuroendocrine tumors. A PET-CT scan was conducted, which showed increased metabolic activity in the left upper arm but no evidence of distant metastasis, suggesting localized disease (Figure 1B).

Histopathological analysis of the excised tumor demonstrated nodular growths in the subcutaneous tissue, composed of cells with pale cytoplasms and inconspicuous nucleoli, a hallmark of MCC (Figure 1C). These cells densely proliferated without evidence of vascular or lymphatic invasion. Immunohistochemical staining was positive for CK20 in a characteristic dot-like pattern (Figure 1D), along with epithelial membrane antigen (EMA), chromogranin A, synaptophysin, and CD56, further supporting the diagnosis of MCC. Additionally, the presence of Merkel cell polyomavirus antibody was confirmed (Figure 1E), which is often associated with MCC. With these comprehensive diagnostic findings, the patient was staged as IIA MCC (T2N0M0). The patient underwent the initial tumor resection. During this surgery, a 2 cm margin was ensured, with the tumor measuring 30×33 mm, surrounding indurated erythema measuring 63×54 mm, and the excision diameter being 108×94 mm. A sentinel lymph node biopsy was not performed. This patient showed no marginal positivity of the tumor or vessel invasion. Chemotherapy has not been implemented as there are no prospective clinical trials demonstrating a significant extension of life prognosis, and no regimens are approved by insurance in our country. Following the first surgery, adjuvant postoperative radiation therapy with 58 Gy (29 fractions) was administered.

Eight months post-diagnosis, the patient experienced a sudden onset of fever and dyspnea during the night, necessitating emergency hospital admission. A diagnosis of COVID-19 was confirmed. Two months following intensive treatment with antiviral therapy, including remdesivir, and systemic corticosteroids, the patient rapidly developed in-transit metastasis along the path between the primary tumor site and the regional lymph nodes (Figure 1F). This complication led to additional surgical interventions and further radiotherapy. A second surgery was performed to address a local recurrence, with a 10 mm margin being taken. After the second surgery, adjuvant postoperative radiation therapy with 39 Gy (13 fractions), 3 Gy per fraction, using 6 MeV electron beams was administered.

Unfortunately, the patient also suffered a cerebrovascular accident (stroke) during his recovery, which required rehabilitation and initiation of dual antiplatelet therapy.

## Discussion

This case highlights a potentially crucial aspect of the COVID-19 pandemic and its impact on patients with existing malignant conditions. The rapid progression of MCC following COVID-19 infection and the treatment in our patient raises questions regarding the role of the virus in modifying the tumor microenvironment. The virus is known to induce a significant alteration in immune system dynamics, including both innate and adaptive responses. Inflammatory cytokines, such as interleukin-6 (IL-6) and tumor necrosis factor-alpha (TNF-α), are elevated during COVID-19 infection and can create an environment conducive to tumor growth and metastasis. COVID-19 induces a shift from STAT1 to STAT3 signaling, enhancing pro-inflammatory responses and cytokine storms [5,6]. This alteration enhances elevated levels of cytokines such as IL-6 and TNF-α to drive cancer progression. COVID-19-associated inflammation can activate the NOD-like receptor family, pyrin domain-containing 3 (NLRP3) inflammasome [7], further contributing to severe disease outcomes in cancer patients [8]. SARS-CoV-2 is known to induce the downregulation of major histocompatibility complex class Ι (MHC-Ι), leading to the impairment of antigen presentation ability [9]. Furthermore, SARS-CoV-2 may also negatively interfere with the function of p53 and Rb tumor-suppressing genes [10], which are responsible for MCC oncogenesis [10], suggesting the direct influences on the development of MCC. These inflammatory responses can support cancer cell survival and proliferation, potentially accelerating the course of MCC. The PET-CT/18F-FDG PET-CT scan is a very useful imaging technique in the whole-body staging of MCC. It is also useful for detecting unexpected recurrences and distant metastatic disease [11]. Therefore, using PET-CT scanning might be useful for detecting recurrent tumors of MCC.

Another significant factor is the use of corticosteroids in the COVID-19 treatment regimen. Corticosteroids suppress the immune system, which may diminish the immune response against existing cancer cells [12]. Merkel cell carcinoma (MCC) is known to be influenced by the host's immune response and sometimes exhibits spontaneous tumor regression [13]. Therefore, an immunosuppressive condition could exacerbate tumor progression. These observations suggest that both the virus and its treatment may influence the development and progression of MCC.

COVID-19 treatment may create conditions that facilitate cancer recurrence, including MCC. The use of corticosteroids, immunosuppressive agents, and JAK inhibitors in the COVID-19 treatment regimen can significantly impact the immune system [14-16], potentially exacerbating tumor progression.

Corticosteroids, commonly used to manage severe COVID-19, suppress the immune system and may reduce the body's ability to effectively respond to existing cancer cells. This immunosuppression can be particularly detrimental in cancers such as MCC, where the host immune response plays a critical role in controlling tumor growth and spread [12].

The use of immunosuppressive agents, such as those for managing cytokine release syndrome or other inflammatory complications of COVID-19, further diminishes the immune system's ability to combat malignancies. This can potentially increase the association with tumor progression [17]. The impact of these immunosuppressive therapies on COVID-19 and other infections has been widely discussed.

JAK inhibitors, which target the JAK-STAT signaling pathway, are used to manage severe inflammatory responses in COVID-19 patients. However, they can also impair immune surveillance and modulation, which are crucial for controlling cancer progression. The disruption of the JAK-STAT pathway can promote a pro-inflammatory environment conducive to tumor growth and metastasis. Consequently, the risk of MCC may increase with the use of JAK inhibitors [18]. This mechanism is well-documented in the context of severe inflammatory diseases.

Additionally, the inflammatory cytokine storm induced by COVID-19, characterized by elevated levels of cytokines such as IL-6 and TNF-α, can create a tumor-friendly microenvironment [19]. This inflammation supports cancer cell survival and proliferation, potentially accelerating the progression of MCC and other cancers. The role of cytokine storms in severe COVID-19 cases has been extensively studied.

The use of corticosteroids and other immunosuppressive treatments during COVID-19 therapy has been linked to an increased risk of cancer recurrence. Corticosteroids, often administered to manage COVID-19 complications, have strong immunosuppressive effects that can impair the body's ability to mount an effective anti-tumor response. Studies indicate that glucocorticoid therapy, particularly when started early in immune checkpoint inhibitor treatment, is associated with decreased overall survival and progression-free survival in cancer patients [20].

## Conclusions

The observations from this case highlight the need for a comprehensive approach to managing cancer patients during COVID-19 infection and treatment. Although the correlation between the clinical course and COVID-19 remains ambiguous, this case emphasizes the importance of further research to elucidate the mechanisms underlying MCC development and its potential interactions with COVID-19.
